# Tailoring inkjet-printed PEDOT:PSS composition toward green, wearable device fabrication

**DOI:** 10.1063/5.0117278

**Published:** 2023-01-03

**Authors:** Marina Galliani, Laura M. Ferrari, Guenaelle Bouet, David Eglin, Esma Ismailova

**Affiliations:** 1 Mines Saint-Etienne, Centre CMP, Département BEL, F-13541 Gardanne, France; 2 INRIA, Université Côte d'Azur, 06902 Sophia Antipolis, France; 3 Mines Saint-Étienne, Université Jean Monnet, INSERM, U1059 Sainbiose, Saint-Étienne F-42023, France

## Abstract

Inkjet printing remains one of the most cost-efficient techniques for device prototyping and manufacturing, offering considerable freedom of digital design, non-contact, and additive fabrication. When developing novel wearable devices, a balanced approach is required between functional, user-safe materials and scalable manufacturing processes. Here, we propose a tailor-made ink formulation, based on non-hazardous materials, to develop green electronic devices aimed at interfacing with humans. We demonstrate that developed ink exhibits high-resolution inkjet printability, in line with theoretical prediction, on multiple wearable substrates. The ink's chemical composition ensures the pattern's enhanced electrical properties, mechanical flexibility, and stability in water. The cytocompatibility evaluations show no noxious effects from printed films in contact with human mesenchymal stem cells. Finally, we fabricated a printed wearable touch sensor on a non-woven fabric substrate, capable of tracking human steps. This is a step toward the development of green wearable electronics manufacturing, demonstrating a viable combination of materials and processes for biocompatible devices.

## I. INTRODUCTION

The field of wearable electronics is growing rapidly and is guided by an awareness of users' safety and the environmental impact of electronic products due to their numbers and short life cycle. The materials and manufacturing processes are the principle concerns when adopting green electronics concepts for novel technology development. This is further accentuated in the case of electronic wearables that interface with humans, where, for hygiene reasons, devices are required to be both disposable and biocompatible. With the aim of achieving a sustainable, green approach to designing wearable devices, multiple considerations need to be taken into account at the design stage of the electronics.^1^ For an efficient use of resources, the design and manufacturing of devices need to include an effective recycling pathway, make use of biocompatible and biosourced materials, and result in minimal waste production.

Inkjet printing is a highly efficient, non-contact, additive, solution-based patterning technique with low-cost, high-quality, and high-throughput advantages.^2,3^ Owing to these characteristics, it became the reference method for the manufacturing of functional materials, especially those that are chemically incompatible with other microfabrication processes on flexible and wearable substrates.^4^ Inkjet printing stands out for its high-resolution (10–100 *μ*m) digital patterning while avoiding precious material waste.^5^ As it is a drop-on-demand deposition technique only a small amount (in the picoliter range^6^) of active materials are dispensed to create the design. This process has allowed for the printing of various functional materials and the fabrication of novel wearable devices. Examples range from wearable displays to health monitoring sensors^7,8^ and even green electronic memory cells.^9^

Inkjet printing uses bespoke solution-based materials, specifically formulated to match the process' governing multiplex physics.^10,11^ The optimum balance of the inks' physical and chemical properties yields stable fluid jetting, reduced nozzle clogging, and a uniform dispensed film morphology.^12–14^ Among the wide variety of organic and inorganic printable materials available, conducting polymers are frequently chosen for flexible and bio-electronic devices fabrication. In the last decade, conducting polymers (CPs) have been extensively employed due to their mechanical and chemical properties, enabling their fast, low-cost processability.^15^ Among CPs, poly(3,4-ethylenedioxythiophene):poly(styrene sulfonate) (PEDOT:PSS) is one of the most used p-type semiconductors, both in industry and academics, owing to its high conductivity, chemical stability, semi-transparency, and, most importantly, commercial availability.^16,17^ The commercial PEDOT:PSS is a water dispersion with a solid content of active material typically not exceeding 5%.^18^ The aqueous mixture is blended with additives to fine-tune its chemical, physical, and electrical properties toward targeted applications, while at the same time accommodating the specifics of a particular patterning method. The reported formulations based on commercial PEDOT:PSS have been typically proposed to achieve high-end electrical properties in photovoltaics applications.^19^ Studies describing the role of different additives in the PEDOT:PSS inks, supported by surface tension measurements and high-speed camera imaging, have been mostly centered on printed organic solar cell fabrication.^20^ Moreover, it is possible to obtain a highly conductive inkjet printable PEDOT:PSS in combination with an ionic liquid additive, as shown in elastic interconnect fabrication.^21^ PEDOT:PSS formulations have been used in the printing of textile and paper-based electrophysiology electrodes, for cutaneous sensing. By employing ethylene glycol, organic solvents and a surfactant, the jetting and drying processes of the ink were optimized on such unconventional substrates.^22,23^ Indeed, the inkjet processing is strongly dependent on substrate surface properties. The role of several printing parameters to improve the PEDOT:PSS ink deposition on hydrophobic silicon surfaces, including drop spacing, substrate temperature, and the number of layers, have been thoroughly investigated for solar cell manufacturing.^19^ Therefore, similar studies would be necessary to move this patterning technique from silicon-based, planar flexible devices to novel fields of applications.

With an increase in the adoption of printable materials in the field of bioelectronics and wearable devices, biocompatibility, mechanical robustness, and environmental stability evaluations are often necessary to justify the choice of materials. Several studies demonstrated promising compatibility of commercially available PEDOT:PSS inks with cell cultures,^24–27^ cutaneous contact,^28,29^ and even *in vivo* neural interfacing.^30^ In wearables, adverse reactions can occur at the interface with the body such as tissue irritation, inflammation, or development of a foreign-body response. Concerning synthetic reactivity, organic materials and flexible devices that are composed of carbon are further envisioned for green processing and efficient recycling.^31^ According to chemical datasheets, some components can be considered as non-hazardous, taking into account their exposure levels vis a vis contact with skin, eyes, inhalation, ingestion, acute and chronic contact, and their dosage. A key aspect is to characterize their potential biocompatibility, which requires validation prior to a particular usage scenario. Therefore, this practice is guided by the use of non-hazardous chemicals when bio-interfacing, as well as taking into account the sustainability aspects of manufacturing. The use of green solvents and reagents during synthesis reduces the quantity of toxic chemical waste generated and allows for non-hazardous disposal.

Here, we present an electrically conducting PEDOT:PSS formulation for the inkjet printing of wearable devices. The ink's electrical properties were evaluated in correlation with the specifics of the printability process. The key characteristics were experimentally determined for a broad printability assessment in relation to the theoretical predictions. The mechanical and water stability results indicate an appropriate robustness of the printed designs for diverse wearable applications. With a view to target wearable bioelectronic device manufacturing, the cytotoxicity assays show high human stem cells viability when in contact with the ink-coated substrates. Finally, the formulated ink allows the fabrication of a printed, wearable gait sensor on a paper-like substrate, with a minimal device footprint while precisely tracking walking activity.

## II. RESULTS

### A. Inkjet printable PEDOT:PSS ink formulation

**FIG. 1.**
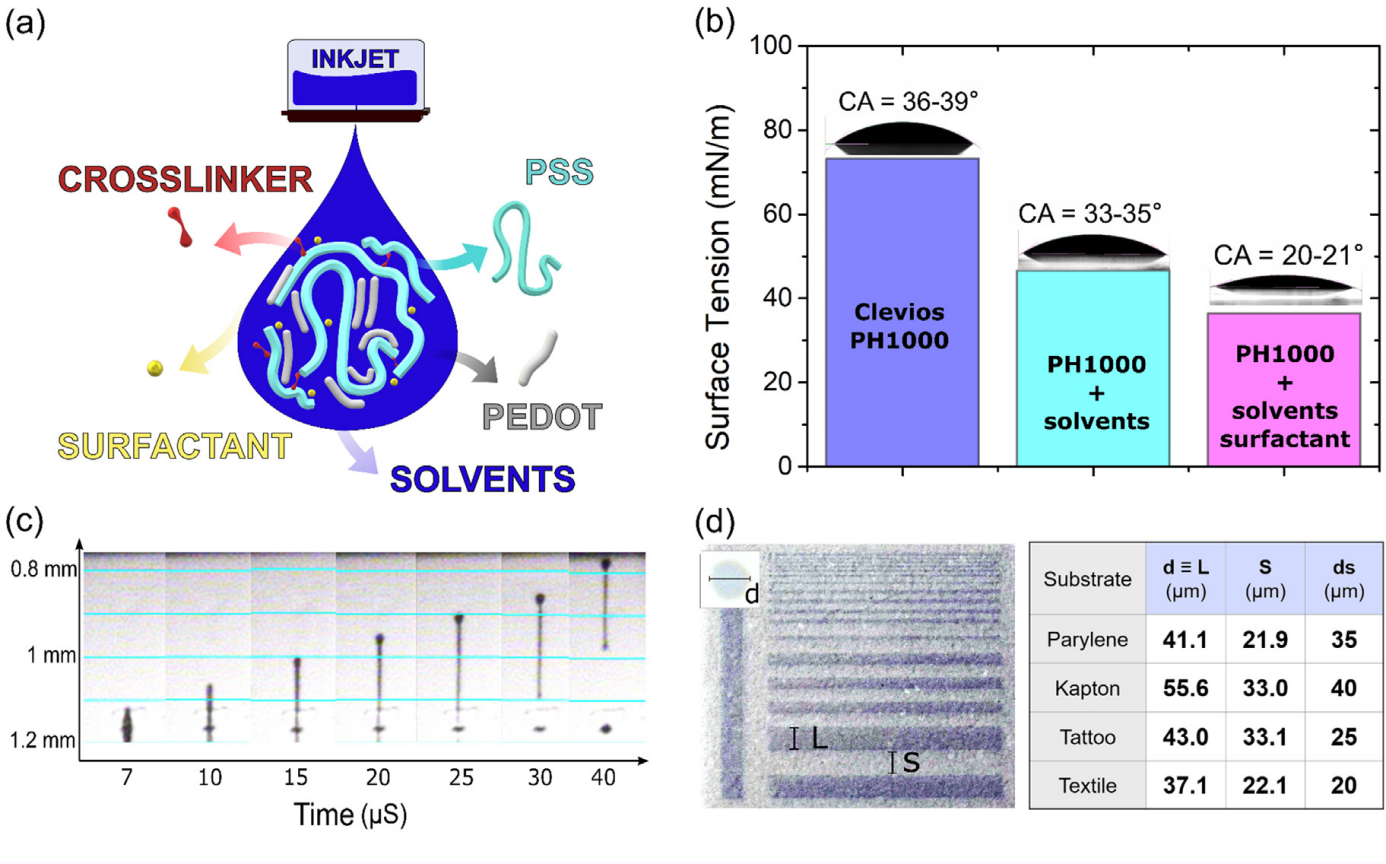
Inkjet printable PEDOT:PSS. (a) Schematic representation of the chemical composition of the ink: the PEDOT and PSS chains in aqueous dispersion, the organic solvents, a surfactant, and a cross-linking agent. (b) Surface tension and contact angle (CA) values of the PEDOT:PSS following the ink formulation steps by adding solvents and the surfactant on glass. (c) Ejected drop timeframes from the nozzle view. The y-axis indicates the drops' traveled distance as a function of time. (d) On the left: resolution pattern printed on parylene C substrate. The top left inset shows an optical image of a single drop for the inspection of the printed drop shape and its diameter *d*. L and S labels indicate the linewidth and the interline space, respectively. On the right: table summarizing the resolutions obtained and adopted drop spacing values for different flexible substrates.

Initially, the commercially available PEDOT:PSS water dispersion (Clevios PH1000 by Heraeus) was used in the ink formulation. To enhance the ink's electrical properties and film forming characteristics, additional chemicals, based on their low hazard indications, were added [Fig. 1(a)]. First, the high boiling point solvent dimethyl sulfoxide (DMSO, b.p. = 189 °C), at the concentration of 10% w/w, was selected as an electrical conductivity enhancer. High boiling point solvents with a high dielectric constant, such as DMSO, can reduce the coulombic interactions between the PEDOT and the PSS chains.^32^ Here, the DMSO is used instead of the typical ethylene glycol, which is also adopted as an equivalent solvent, as it has been shown to provide smooth printing patterns and is a minimally hazardous substance.^21,33,34^ The 10% w/w concentration was selected in order to enhance the films' properties, while at the same time not excessively diluting the solution.^35^ A second solvent, with a low boiling point, isopropanol (IPA, b.p. = 82.5 °C), was added at the concentration of 5% w/w. Mixing high and low boiling point solvents permits the control of the “coffee ring effect” that influences the printed film morphology and ultimately its electrical performances.^36^ Additionally, TWEEN 20 at 0.5% w/w concentration was adopted as a surfactant. Such a small concentration (<1% w/w) has been reported to be sufficient to both lower the surface tension and enhance conductivity.^37^ Finally, the crosslinker (3-glycidyloxypropyl)trimethoxysilane (GOPS) at 1% w/w completed the formulation. The GOPS is a nonvolatile additive that provides the film's mechanical integrity in aqueous media. A film with a cross-linked network and improved adherence to the substrate results from the chemical bonds formed between the GOPS epoxy rings with both the PSS chains and the substrate.^38,39^ It is important to point out that all the chemicals added up to this point are labeled as non-hazardous, with the exception of GOPS. Nevertheless, GOPS' reactivity should be suppressed by the formation of stable siloxane bonds once added to the aqueous-based ink. Moreover, since the GOPS's methoxysilane groups are highly reactive when in contact with water,^40^ a washing step to remove from the specimen any excess of eventually unreacted GOPS molecules from the printed samples before any usage represents an effective measure.

### B. Theoretical ink printability evaluation

To evaluate the ink's printability, the surface tension, density, and viscosity were measured. The surface tension of the initial Clevios PH1000 was 73.21 mN/m [Fig. 1(b)], which is close to the value of water (72.8 mN/m at 20 °C). After adding DMSO and IPA, it decreases to 46.6 mN/m. Finally, the addition of TWEEN20 provoked a decrease to 36.43 mN/m, which is within the surface tension target range for the printer (28–42 mN/m) used in our work.^6^ The surface tension variation was visually confirmed by the decrease in the contact angle, measured on a clean glass substrate, that started from 36° to 39° and reaches 20°–21° [Fig. 1(b)]. The ink density and viscosity were found to be 0.99 g/cc and 25 mPa, respectively. An important issue when formulating an ink is to achieve stable fluid jetting, reduced nozzle clogging, and a uniform printed film morphology. This is evaluated via the adimensional Z number [Eq. (2)], which is obtained by calculating the Reynold number—the ratio between inertial forces and the viscous forces [Re, Eq. (2)], the Weber number [We, Eq. (3)], and the ratio of inertial forces over the surface forces.^41^ Based on these values, the ink's printability is theoretically determined by calculating the Z number, as the ratio between Reynold number and Weber number

$$Z=\frac{Re}{\sqrt{We}},$$ (1)

$$Re=\frac{\nu \rho d}{\eta },$$ (2)

$$We=\frac{\nu^{2\;}\rho d}{\sigma },$$ (3)

where *ρ* represents the ink density, *η* represents the ink viscosity, *σ* represents the ink surface tension, *d* represents the cartridge nozzle diameter, and *v* represents the velocity of the jetted drop.

A *Z* value between 1 and 10 is defined as printable.^41,42^ The calculated *Z* number of the formulated ink here is 1.1, and is thus considered suitable for inkjet printing.

### C. Droplets characteristics and resolution

Printing quality depends on numerous parameters controlling optimization of droplet velocity and shape. The highest quality results reported here were obtained with a firing voltage of 24 V and the printing waveform found in the supplementary material Fig. S1. In all the experiments, the ink cartridge was kept at room temperature (19–22 °C) to reach reproducible ink rheometry. After visual analysis of the droplet formation [Fig. 1(c)] and using timeframes data, the drop velocity was estimated to be in the region of 10 m/S. In the image, a typical ink column thins and elongates during its falling trajectory. The ink takes the shape of a well-defined spherical drop with a so-called ligament and does not form satellite droplets.

The resolution assessment of the printed patterns was performed on different flexible substrates that are widely used in bioelectronics, namely, parylene C, polyimide Kapton foil, temporary tattoo paper, and a fabric.^43–45^ Figure 1(d) left shows the test pattern print on parylene C. These diverse substrates possess distinct surface energies and absorption properties, affecting the drops' spreading. The interplay between the ink surface tension and the substrate wettability results in printed drops of varying diameters. The drop diameter influences the choice of the printing drop space, and these aspects are both correlated ultimately to the printing uniformity. As a rule of thumb, to get overlapping drops, the drop spacing has to be smaller than the droplet diameter. We observed drop diameters of 41.1, 55.6, 43.0, and 37.1 *μ*m on parylene C, polyimide, tattoo paper, and fabric substrates, respectively. The printing resolution is defined as the minimum space between two separated lines [S value in Fig. 1(d)]. When printing on parylene substrate, well-defined printed lines are obtained by employing a drop spacing of 35 *μ*m, achieving a resolution of 21.9 *μ*m. However, when printing on polyimide foil, 40 *μ*m was found to be the optimal drop spacing [Fig. S2(a)] obtaining a 33.0 *μ*m resolution. This confirms that polyimide has a higher surface energy than parylene C. In the case of tattoo paper, while an average drop diameter of 43 μm [Fig. S2(b)] was obtained, a drop spacing of 25 *μ*m is necessary to guarantee a uniform film with a resolution of 33.1 *μ*m. This is due to the tattoo paper's surface roughness,^44^ which requires greater material deposition to produce a continuous line pattern. Finally, when printing on fabrics, an optimal drop space of 20 *μ*m is needed to generate continuous conducting lines with a resolution of 22.1 *μ*m [Fig. S2(c)]. Notably, when using fabrics, ink absorption occurs requiring the use of the smallest drop spacing possible. Altogether, these outcomes indicate that the best resolution is obtained on parylene C and fabric substrate (∼22 *μ*m). Overall, the PEDOT:PSS ink allows for fine patterning on all the substrates examined, providing an average printing resolution of 27.5 ± 4.5 *μ*m, which is well-suited for miniaturized organic electronic device fabrication.

**FIG. 2.**
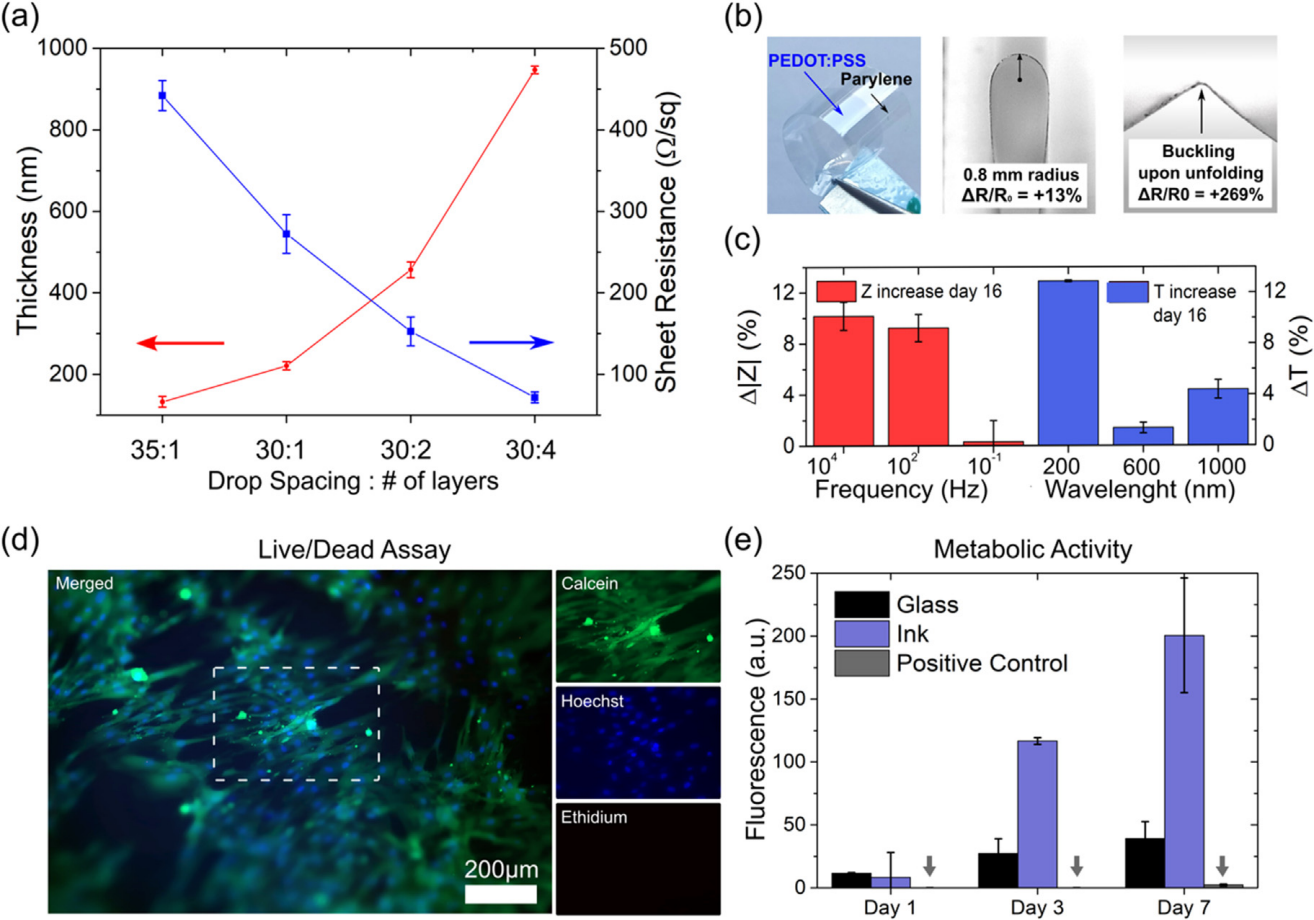
Formulated PEDOT:PSS ink characterization. (a) Printed film thickness vs sheet resistance as a function of the drop spacing and layer number. 1 cm^2^ samples are printed employing a drop spacing of either 35 or 30 *μ*m and by depositing one, two, or four layers. (b) Photographs of the sample used for bending stress experiments. From left to right: picture of thin PEDOT:PSS film (35 *μ*m drop spacing, one layer) printed on parylene C substrate (10 μm thick); this sample when bent at a 0.8 mm curvature radius; this sample after it undergoes a complete folding stress, showing a buckle along the folding line. The ΔR/R0 values indicate the normalized samples' electrical sheet resistance increase, over three samples, as a function of the bending curvature radius and cycles. (c) Ink stability in aqueous environment: EIS and UV-Vis spectroscopy results after 16 days of sample storing in water, the transmittance is reported for 200, 600, and 1000 nm wavelength values, and impedance modulus is reported for 0.1, 100, and 10 kHz frequencies. (d) Fluorescence imaging of live/dead staining performed after 7 days of hMSCs cultured on ink-coated glass. Images of calcein (live), Hoechst (nuclei), and ethidium (dead) stainings are on the left. The dashed rectangular frame represents the zoomed area for the individual staining images (on the right). (e) Presto Blue assay performed on hMSCs cultured on top of glass slides, either coated with ink or not, for 1–7 days (n = 3). The positive control (dead cells, marked with arrows) corresponds to cells seeded on a glass slide and further treated with 70% methanol. The error bars in (a), (c), and (e) correspond to the standard deviation over three measurements.

### D. Characterization of printed PEDOT:PSS films

#### 1. Electrical properties

The characterization of electrical properties for printed films was studied as a function of the drop spacing and number of printed layers [Fig. 2(a)]. As expected, the film thickness and its sheet resistance were found to be inversely proportional. Printing at 725 dpi (∼35 *μ*m drop spacing) resolution with 10pl nominal drop volume cartridge resulted in films with thicknesses of 132 ± 13 nm and a measured sheet resistance value of 442 ± 18 Ω/sq. By decreasing the drop spacing to 30 *μ*m, thicker films were obtained (221 ± 10 nm) with a lower sheet resistance (272 ± 23 Ω/sq). Consecutive printing of layers can further increase the film thickness. Two layer printed films are almost double in thickness (456 ± 19 nm) and lower the sheet resistance value by half (152 ± 17 Ω/sq). The same relationship is observed when four layers are deposited (947 ± 9 nm, 71 ± 6.6 Ω/sq). These results are invariant with respect to the used cartridge. Films printed with smaller nominal drop volume cartridges (2.4 pl) followed the same trend for one and two layer films (supplementary material Fig. S3).

**FIG. 3.**
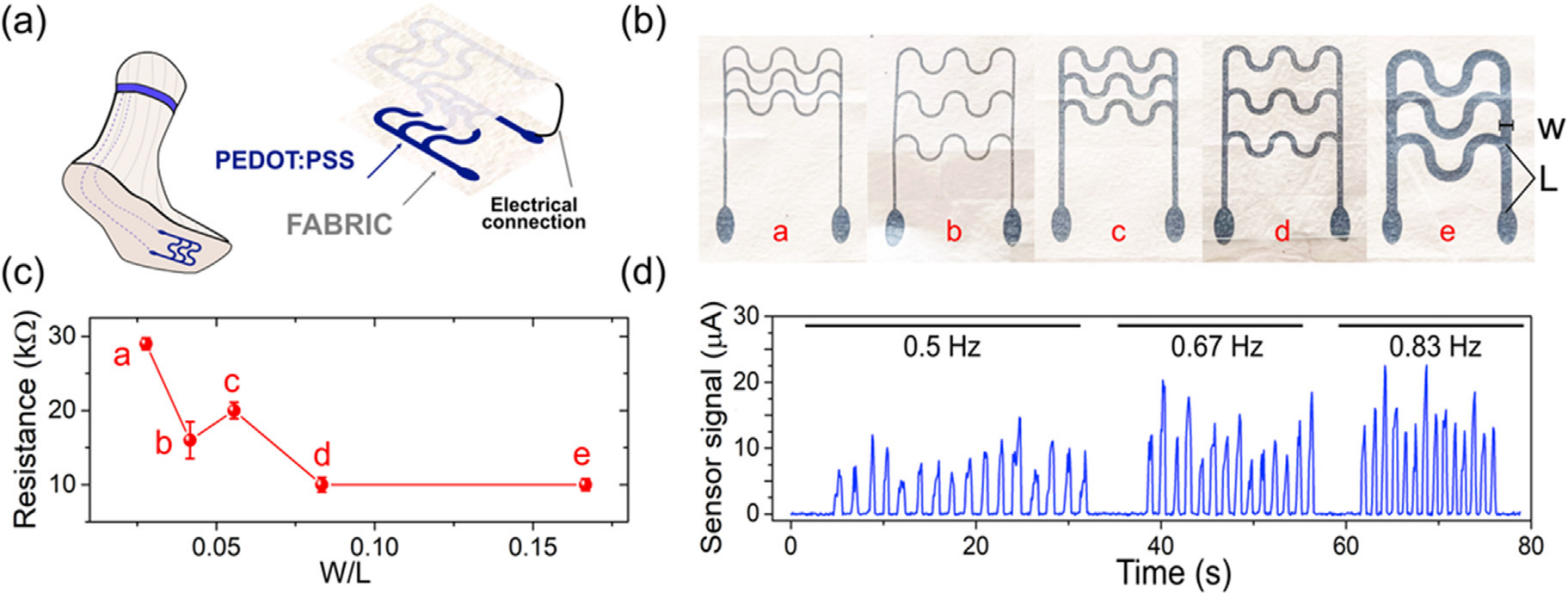
Printed wearable step tracker. (a) The assembled bilayer sensor made of two facing serpentine electrodes electrically connected. Step tracker schematic once integrated into a sock. (b) Pictures of five sensor designs with distinct W/L ratios, where W and L indicate the printed lines width and the distance between the first serpentine and the oval-shaped connector, respectively. (c) Electrical resistance values associated with each of the designs labeled by the W/L ratio value. Error bars represent the standard error over three measurements. (d) Sensor's data recordings at 0.5, 0.67, and 0.83 Hz step frequencies that correspond to 60, 80, and 100 steps/minute, respectively.

#### 2. Mechanical properties

Mechanical stability evaluations were performed via a bending stress test of printed films while measuring their sheet resistance. In this way, it is possible to detect potential film damage (e.g., cracks, delamination) responsible for the loss of electrical performance occurring after extensive use. For this test, a line (0.5 × 70 mm^2^) was printed on a thin parylene C substrate (10 *μ*m thick) [Fig. 2(b), left] that is considered as a highly flexible substrate. In the literature, a bending radius of 1 mm has been reported as the mechanical threshold for flexible electronics materials.^46^ In this study, we set a bending radius to 800 *μ*m [Fig. 2(b), center]. The film's electrical resistance variation (ΔR%) is reported as the difference between the initial value and that measured after the bending experiment was concluded. Our formulated ink showed a lower film sheet resistance variation (+13%, from 64 ± 7.7 to 72 ± 10 kΩ/cm) after 5000 bending cycles, compared to the commercial Pjet 700 ink (+17%, from 72 ± 2.9 to 84 ± 11 kΩ/cm). The samples retained appreciable electrical performances, and under optical inspection, they do not show any visible damage such as cracks or delaminated fragments. Moreover, our printed sample was further folded to reach 0 mm bending radius for 10 cycles. As expected, a considerable increase in resistance to 269% ± 29% was observed from such an intense mechanical stress. The test sample was also observed to have developed a buckling at the fold point in this case [Fig. 2(b), right]. When parylene C film is similarly tested, a crack is observed at the fold point (data not shown). Therefore, the crease can be attributed mainly to substrate limitations. These outcomes confirm the formulated ink's robust mechanical properties measured at the lower limit of the bending radius and that are comparable with PEDOT:PSS-printed electrodes, which show a sheet resistance increase in 25% after 600 bending cycles at a 5 mm bending radius.^47^

#### 3. Ink stability in aqueous environment

In order to evaluate the ink stability in water, electrochemical impedance spectroscopy (EIS) and UV-visible transmittance spectra tests were performed on printed samples before and after immersion in water at constant ambient temperature for 16 days. This method allowed for investigation into material loss occurring due to a possible degradation of PEDOT:PSS-printed device during usage. Figure 2(c) shows the increase in percentage impedance modulus and the transmittance value, respectively. The EIS results, shown in Fig. 2(c), red, give an overall increase in the impedance of less than 10%. A major variation (10.17% ± 1.09%) is found at the highest frequency (10 kHz), and a much less significant variation (0.3% ± 1.65%) is observed at the lowest frequency (0.1 Hz). Considering that, the film's electrical behavior can be modeled as that of a resistor and a capacitor below and above the 100 Hz frequency range, respectively.^48^ Thus, indicating that the major changes are related to a resistive circuit component. Consistent with these results, we observed a limited increase in the film's transmittance, with its maximum at 200 nm (12.8% ± 0.06%) and its minimum at 1000 nm (4.39% ± 0.73%). This increase in transmittance was due to some material loss that can occur when some of the printed film dissolves in water. It is known that PEDOT:PSS swells in contact with water as a result of the deprotonation of the sulfonate groups in its PSS-rich regions,^49^ eventually promoting small molecule migration from the film. The addition of GOPS is known to enhance the stability of PEDOT:PSS film^38^ and therefore, as a consequence, we observe small variations in the measured impedance and transmittance.

#### 4. Cytocompatibility assay

Ink suitability for bio-interfaced devices is evaluated by performing live and dead imaging and a resazurin-based assay, an indirect metabolic activity assays, on human mesenchymal stem cells (hMSCs). These cells, previously used in 3D printing of artificial skin patches, were seeded onto ink-coated glass substrates.^50,51^ The evaluations showed a high cell viability (>99%) compared to the staining methods as a control test (see Fig. S5), based on negative (glass) and death controls (uncoated glass substrates further treated with 70% methanol). During experiments, almost no dead cells were noticed, and a growing number of alive cells were seen from day 1 to day 7 in cultures interfacing with the ink. This is further described in the control experiments demonstrated in Fig. S6, and, as expected, the positive control showed only dead cells due to the methanol present. Figure 2(d) shows representative fluorescence images of live/dead staining performed after 7 days of culture merging calcein AM, ethidium homodimer III, and the Hoechst markers showing live cells in green, dead cells in red, and the cell nuclei in blue, respectively. A resazurin-based assay, as an indicator of cells viability through their ability to reduce the dye reagent content, was performed, and its results are reported in Fig. 2(e). Interestingly, after 1 day of culture, the rate of resazurin reduction by metabolically active cells measured by fluorescence on the glass controls and on the ink-coated samples were equivalent. After 7 days of culture, the ink-coated samples show a five times higher fluorescence (p = 0.004) than both controls. Due to the absence of dead cells in the live/dead imaging, we concluded that the high fluorescence corresponds to the metabolic activity of living cells, consistent with the cell growth observed in live/dead experiments. Therefore, no signs of cytotoxicity were observed from cells interfaced with the formulated ink up to 7 days, indicating potential biocompatibility of the developed devices using our ink.

### E. Printed wearable step tracker

#### 1. Sensor working principles

The sensor is made of two facing serpentine electrodes that are printed on melt-blown non-woven polypropylene fabric [Fig. 3(a)]. It is designed so when the two electrodes are touching each other a closed electrical circuit is created. In step tracking applications, it can measure a closed (stance phase) or open (swing phase, when the foot is in the air) circuit states. This alternance between states allows the calculation of the step frequency and thus a walking rate calculation capability. Multiple designs, by varying the sensor's linewidth (W) and the gap between the first serpentine and the connector (L), were evaluated. By combining different W and L dimensions, five designs were created with distinct W/L ratios [Fig. 3(b)]. The electrical resistance for each design is indicated by the W/L ratio value in [Fig. 3(c)]. The different values follow a design-dependent rational. In the reported configurations, the current flowing between the two connectors' passes via the shortest path, which is the first serpentine from the bottom. A lower W value defines narrower tracks, thus a circuit with higher resistance. In contrast, a lower L distance defines a shorter path for the current, thus resulting in a lower resistance. Design *e* in [Figs. 3(b) and 3(c)], which has a W/L ratio of 0.16 corresponding to a W and L equal to 2 and 12 mm, was selected for future sensor evaluations. As it has less resistance, the sensor is able to accommodate some misalignment between the two electrodes during the gait, due to the higher W.

#### 2. Sensor's performances

To assess the sensors' performance, a weight was periodically placed on the sensor surface mimicking a step. When the weight is applied, the sensor becomes a closed circuit and the electrical response is recorded in *μ*A. [Fig. 3(d)] shows the sensor recordings at three different step rates. Considering the signal from one foot, the sensor resolves walking frequencies of 0.5, 0.67, and 0.83 Hz, that correspond to 60, 80, and 100 steps/minute, respectively. In the recording, in [Fig. 3(d)], a 0 *μ*A output represents the swing phase, while a signal in the range of 5 and 25 μA represents the stance phase. The sensor's ability to distinguish between slow and moderate touch rates, validating its utility as a human pedometer. Analyzing the signal amplitude, we can extrapolate positional information related to the plantar pressure. The variability of the current peak amplitudes observed in each step frequency recording is due to a diverse pressure configuration on the sensor. Lower amplitude signals (6.31 *μ*A ± 0.58) correspond to a foot pressure distributed on the upper serpentine element of the sensor. On the other hand, when the pressure involves the lower serpentine elements, a higher amplitude peak is recorded (20 *μ*A ± 1.17).

## III. DISCUSSION

The addition to the DMSO, IPA, TWEEN20 surfactant, and the GOPS cross-linking agent to the PEDOT:PSS dispersion resulted in a performant, printable ink formulation. As reported, the DMSO enhances the electrical conductivity by improving polymeric chains morphological rearrangement during the film drying process.^52^ Indeed, in commercial PEDOT:PSS water dispersion, an excess of PSS chains is added, both to act as a counterion for the PEDOT+ chains and to electrostatically stabilize them in an aqueous suspension. The PSS has an insulating nature; thus, the excess of PSS increases the overall material electrical resistance. DMSO reduces the electrostatic inter-chain interactions between the PEDOT rich grains and the insulating PSS rich shells. In the deposited film, this contribution results in the coarsening of PEDOT domains, which, in turn, results in the formation of more conductive pathways, ultimately facilitating the inter-PEDOT chains' charge transport.^53–55^ On the contrary, the IPA's low boiling point solvent controls the so-called “coffee ring effect.” When a drop is dispensed onto a substrate, the solvent's evaporation occurs faster at its edges. Capillary forces then drag the ink toward the drop boundaries, resulting in migration toward the drop boundary. Thus, leading to excess deposition and solidification of the ink solute at the border.^12^ The combination of low and high boiling point solvents avoids inhomogeneities in the dried film, changing the shape of solute deposition from a circumferential, ring-like pattern to a solid dot-like shape.^36^

The addition of TWEEN20 was also crucial to optimization of the ink formulation, since surfactants help in improving film homogeneity by reducing the fluid surface tension.^37^ A homogeneous coating occurs when the surface energy of the substrate has a higher value than the surface energy of the solution, yet not too different. In this respect, surfactant molecules reduce the surface tension to a value closer to the surface energy of the substrate, and enhance crystallization of PEDOT chains due to the weakening of the ionic interactions between PEDOT and PSS.^37^ By depositing defined patterns with appreciable qualities onto different substrates, the beneficial role of the aforementioned additives throughout the full printing process was demonstrated. Additionally, it allowed for optimal inkjet printing parameters to be defined.

A performant ink must be stable under air exposure, high humidity circumstances, and long-term water immersion with negligible conductivity decay or structure deterioration.^56^ Therefore, we investigated the conductivity of the printed film with respect to its thickness and then assessed the film's flexibility in terms of bending capability as well as stability in aqueous environment. The mechanical cycling stress tests conducted on the ink revealed appreciable intrinsic electrical stability under tension strain, a highly required characteristic in flexible electronics applications. This means that an excellent film adhesion is developed at the interface with the substrate. Additionally, the deposited ink layers showed adequate water stability over time, exhibiting good retention of electrical properties and appreciable adhesion to the substrate under wet conditions.

Therefore, the combination of inkjet printing, which has been reported as a deposition method that enhances the PEDOT:PSS films' water stability,^25^ together with GOPS, represents a promising approach to characterize the film properties lost in water over time in wearable conditions. Overall, both the impedance and the film transmittance confirm the stability of our ink in contact with water. Remarkably, the interplay of the employed additives play a role in controlling and enhancing the ink performance. In addition, their nontoxic nature confers the material with promising qualities for bio-interfaced device fabrication. Indeed, the preliminary cytocompatibility evaluations do not reveal any noxious effects on human mesenchymal stem cells, yet not fully implying its non-hazardless. Further experiments will be required to assess the complete biocompatibility of the fabricated devices in accordance with specific regulations. Finally, the ink deposition on off-the-shelf melt-blown fabric resulted in an effective method for a one-step gait sensor fabrication, which is directly transferable to a variety of substrates according to the application needs. The embedding of soft conducting materials onto fabric is an established approach to obtain electronic textiles in the shape of smart garments employed for a variety of physical and biochemical body parameter monitoring.^22,57,58^ The sensor shows great potential to distinguish between slow and fast walking rates, providing information on the user's walking activity and insights on the plantar pressure distribution. Such spatial information can be translated, after extended calibration, into a foot pressure map.

## IV. CONCLUSIONS

We have presented an effective methodology to formulate and characterize an inkjet printable PEDOT:PSS formulation from a commercial solution all the way to a wearable device fabrication. We theoretically evaluated the ink's printability and showed experimentally high-quality ink deposition on some of the most used substrates in bio- and flexible electronics. We show here how to balance and optimize the interactions between the materials and the inkjet printing process. Indeed, the ink's chemical composition resulted in a material with enhanced electrical properties, mechanical flexibility, and water stability that are particularly interesting for wearable bioelectronic devices. The ink formulation approach can be easily translated to any water-soluble conjugated polymers. The use of known chemicals resulted in an ink that is cytocompatible. The wearable step tracker, fabricated through the patterning of the formulated ink onto a paper-like fabric substrate, shows great potential to be seamlessly integrated into wearables, such as shoes or socks. The developed ink offers high applicability and versatility in disposable electronics, conformable biomedical devices, and green flexible sensors.

## V. MATERIALS AND METHODS

### A. Ink formulation

The ink consists of commercially available PEDOT:PSS water dispersion Clevios PH1000 (Heraeus) 10% w/w of DMSO (Merck), 5% w/w of IPA (Merck), and 0.5% w/w of TWEEN20 (Merck). The solution was mixed for 20 min in an ultrasonic bath. The ink was kept in the fridge at 4 °C. Just before printing, 1% w/w of (3-glycidyloxypropyl)trimethoxysilane GOPS (Merck) was added.

### B. Ink rheology

The surface tension of the solutions is evaluated at constant ambient temperature with an optical contact angle measuring unit and a contour analysis system (Apollo Instrument, OCA200) via the pendant drop method. The same tool was used to measure the solution's contact angle on a glass substrate. The ink viscosity was measured by scanning the shear rate from 1 to 1233 $s^{-1}$, and the actual value was taken at 1000 $s^{-1}.$^59^

### C. Droplets characteristics and resolution

Prior to printing, the ink was filtered with a 0.2 μm cellulose acetate filter. We employed Dimatix Fujifilm DMP 2800 Inkjet printer (Dimatix Material Printer, Fujifilm Dimatix, Santa ovenClara, CA, USA) with 10 and 2.4 pl nominal drop volume cartridges.

The flexible substrates used for characterization were parylene C vapor deposited film (SCS Labcoter), polyimide Kapton foils (ADDEV Materials), temporary tattoo paper (Silhouette America, Inc, US), and a commercial non-woven fabric. Before printing, only parylene C and Kapton substrates were surface-treated, through a mild O_2_ plasma treatment at 50 W for 1 min (PE100—Plasma Etch, Inc). We performed the ink curing process, of those printed on the parylene C and Kapton samples by placing them on a hot plate at 130 °C for 10 min, while we cured the tattoo paper and fabric samples in an oven at 60 °C for 1h. Before any further characterization, we washed the samples with de-ionized water to remove the excess of PSS chains, potentially not-crosslinked molecules and unreacted GOPS fraction, and then dried the samples with nitrogen gas. To investigate the achievable resolution, we inspected the resolution patterns printed on different substrates through images obtained with an optical microscope (Nikon Eclipse L200).

### D. PEDOT:PSS film characterization

#### 1. Electrical properties

To electrically characterize the ink, we printed one to multiple layers of a squared design (area 1 cm^2^) on a polyimide Kapton substrate. We measured the film thickness with a mechanical profilometer (AMBIOS technology XP-2) and the sheet resistance through a four-point set-up (Keithley source measure unit).

#### 2. Mechanical properties

We assessed the formulated ink performance while undergoing dynamic bending stress with a push to flex bending setup. For this characterization, samples were prepared as follows: Stripes with the size of 50 × 5 mm^2^ made of one layer of PEDOT:PSS ink were printed on a thin parylene C film (10 *μ*m thick) supporting substrate to minimize the substrate's mechanical properties impact on the experiments' outcomes; then the ink was cured on a hot plate at 130 °C for 10 min. The test sample was clamped at its extremities to two plates; its flat position was set as the position 0. Then the plates were moved closer until the sample was bent with a curvature radius of 0.8 mm, which was set as the position 1. A bending cycle consists in moving the plates back and forward between 0 and 1 position. For each sample, we performed 5000 bending cycles and monitored the sheet resistance of the PEDOT:PSS printed film connecting electrically the sample to a Keithley source measure unit. In this setup, schematized in Fig. S4, the conductive PEDOT:PSS film underwent elastic tension strain.

#### 3. Stability in water

For the electrochemical impedance spectroscopy (EIS), we immersed 1 cm^2^ printed glass samples in a phosphate buffered saline (PSB) connecting it to the working electrode of a potentiostat (Metrohm Autolab, Nova 2.1). As the reference and counter electrodes, we used Ag/AgCl and a platinum wire, respectively. We computed the impedance with the potentiostatic mode (0.01 V, 0.1–100 kHz).We evaluated the ink's water stability by monitoring the dry film transmittance with a spectrophotometer (Shimadzu UV-2600) after storing the samples in de-ionized water for 16 days and vacuum-dried it before performing measurements.

#### 4. Cytocompatibility assay

Cell culture: Human mesenchymal stem cells (hMSCs, Lonza, Switzerland) were expanded in a T75-flask to passages 5 (Presto Blue assay) and 7 (live/dead assay from Thermo Fisher Inc.) in α-MEM medium (Sigma) supplemented with 10 vol. % fetal bovine serum (Gibco), 1 vol. % glutamine (Glutamax Gibco), and 1 vol. % penicillin/streptomycin (Sigma). Cells were incubated at 37 °C in a humidified atmosphere containing 5% ${\mathrm{CO}}_{2}$, and the medium was changed twice a week. Before confluence, the hMSCs were seeded at a density of 6200 cells/cm^2^ on the materials (previously sterilized with 70% ethanol for 15 min) inside the wells of a 24-well plate. Tested materials were ink-coated glass coverslips (1 cm). The ink was deposited by drop casting, and it was cured at 130 °C for 15 min. The positive control consisted of dead cells, was produced by seeding cells on the glass substrate and further treating them with 70% methanol (in media as recommended by the manufacturer) for 30 min just before performing the Presto Blue or the live/dead assay. The negative control (all cells alive) consisted of the same seeded glass substrate, but without the methanol treatment. Materials were assessed in triplicate (n = 3).

Metabolic activity: Before the assay, materials were moved to a new 24-well plate. Presto Blue reagent (Invitrogen) was then added to the wells at a concentration of 10 vol. % after 1, 3, and 7 days of culture. Fluorescence was measured (Fluoroskan Ascent, Thermo Scientific) at 590 nm (excitation 530 nm). Fluorescence intensity obtained for the samples was then corrected by subtracting the fluorescence obtained from the equivalent blank controls (same materials cultured under the same conditions, but no cells were seeded on them). Statistical analyses were performed with Jamovi software. Comparisons between materials were made with the non-parametric Student's t-test.

Live/dead imaging: A staining solution containing 2 *μ*M ethidium homodimère III (Interchim), 4 *μ*M calcein AM (Molecular Probe), and 1 *μ*g/ml Hoechst (Thermo Scientific) in PBS (Sigma) was added to the culture wells. After 40 min of incubation, cells were rinsed three times with PBS and further observed under a fluorescence microscope (Zeiss Axio Vert.A1). Excitation wavelengths were 385, 475, and 555 nm for Hoechst, calcein, and ethidium stains, respectively.

### E. Printed wearable step tracker

#### 1. Sensor fabrication

The wearable sensor was fabricated by printing five layers of the digitally drawn design onto commercial melt-blown fabric, considered as a non-woven fabric that has similarity with compact fibrillar texture of paper structure, without any substrate conditioning. The sensor was placed in an oven at 60 °C for one hour to allow for the complete evaporation of the ink's solvents. The electrical performances of different sensor layouts were investigated with a multimeter. The fabrication process can be replicated on different paper-like substrates including papers destined for recycling.

#### 2. Sensor performances

The printed sensor's performance was investigated by facing two equal serpentines electrodes, one in front of each other. Each of the electrodes was electrically connected to an electrical source measurement unit (National Instruments USB-6251 BNC) through oval-shaped interconnections, see Fig. 3(a). The measurement unit recorded the current passing through the two serpentine electrodes as the output signal of the sensor's system. To simulate the gait, we placed a weight (3 kg) onto and away the sensor at three frequencies (0.5, 0.67 , and 0.83 Hz). The weight laying onto the sensor simulated the pressure occurring when the foot touched the ground, while the weight being lifted simulated the foot lifted from the ground during the gait.

## SUPPLEMENTARY MATERIAL

See the supplementary material can be downloaded at: it includes the details on the set jetting waveform used in this manuscript (Fig. S1), the visualization of printing resolution on flexible substrates (Fig. S2), the film thickness vs sheet resistance variation by printing with 2.4 pl nominal drop volume cartridge (Fig. S3), the push to flex sample bending setup schematics (Fig. S4), the control experiment for the hMSCs staining method confirmation (Fig. S5), and representative images of live/dead staining (Fig. S6).

## Data Availability

The data that support the findings of this study are available from the corresponding author upon reasonable request.
